# Unprovoked Submassive Saddle Pulmonary Embolism in an Adult Male After Pfizer COVID-19 Vaccination

**DOI:** 10.7759/cureus.27717

**Published:** 2022-08-05

**Authors:** Blaire D Borisoff, Katherine D Bohn, Justin Sager, Barbara L Gracious

**Affiliations:** 1 Department of Family Medicine, Edward Via College of Osteopathic Medicine, Auburn, USA; 2 Department of Family Medicine, Edward Via College of Osteopathic Medicine, Spartanburg, USA; 3 Department of Family Medicine, HCA Florida Orange Park Hospital, Orange Park, USA; 4 Department of Psychiatry, HCA Florida Orange Park Hospital, Orange Park, USA

**Keywords:** saddle pulmonary embolism, unprovoked pulmonary embolism, pfizer covid-19 vaccine, thrombectomy, deep vein thrombosis

## Abstract

Pulmonary emboli (PEs) occur when the pulmonary artery is blocked by foreign material. In one such instance, this foreign material can be a blood clot that may occur from patient risk factors inducing a prothrombotic state. The relationship between COVID-19 vaccines and a prothrombotic state is novel and changing as our understanding of the relationship between the two evolves. The patient in this case study presented with unrelenting and progressive dyspnea, tachycardia, and unilateral lower extremity swelling two days after receiving the second dose of the Pfizer COVID-19 vaccine. After diagnostic testing, the patient was found to have a submassive saddle pulmonary embolism with subsequent right heart strain. This patient was treated with appropriate anticoagulation therapies, including heparin and apixaban, as well as thrombectomy, and made a complete recovery. The possible relationship between COVID-19 vaccines and thrombotic events supports the need for increased awareness of a potential new risk factor behind the development of PE. It is our hope that this case report will help raise awareness of an association despite the lack of incident data at this time.

## Introduction

Pulmonary emboli (PEs) can occur in prothrombotic states and signify clotting material partially or fully blocking the pulmonary artery or one of its branches. They are usually caused by the combination of Virchow’s triad and other risk factors coming together. Virchow’s triad consists of a hypercoagulable state, some type of endothelial injury, and the presence of venous stasis. Other risk factors can include non-modifiable genetic factors, such as factor V Leiden, or acquired and otherwise modifiable factors, such as immobilization, obesity, or heavy smoking [1]. PEs can be classified based upon the presence or absence of symptoms, their location, and the patient’s hemodynamic stability. PEs can present with a multitude of symptoms that can include anything from an asymptomatic state to sudden death. The most common symptoms reported include dyspnea at rest or with exertion, calf or thigh pain and/or swelling, pleuritic pain, and cough [2]. PEs are named after the location in which they start to block the flow of blood. This can include the lobar, saddle, or segmental branches of pulmonary arteries. Saddle pulmonary emboli are located at the bifurcation of the main pulmonary artery and continue to extend into both the right and left main pulmonary arteries [3]. The diagnosis of a pulmonary embolism is further broken down into massive, submassive, and low-risk categories. These categories are defined by hemodynamic stability, specifically the presence or absence of hypotension and right ventricular dysfunction [4].

The diagnosis of a PE is based on the clinical picture and confirmed by CT pulmonary angiography (CTPA) and D-dimer values. D-dimer values > 500 ng/mL obtained from a quantitative rapid enzyme-linked immunosorbent assay (ELISA) are considered to be positive [5]. A positive D-dimer by itself is not enough to formally diagnose a PE; imaging is needed as well. CT pulmonary angiography (CTPA) is the imaging modality of choice and, when combined with a positive D-dimer, can effectively diagnose a PE with a sensitivity and specificity of 90% and 95%, respectively [6]. A bilateral lower extremity ultrasound with Doppler is another form of diagnostic imaging that assists in diagnosing PE. This form of imaging is highly suggestive if both the ultrasound and Doppler are positive with clinical symptoms of PE also present. However, this form of imaging has a low sensitivity since the thrombus in the lower extremities might have already moved into the lungs when the ultrasound is performed [7]. Electrocardiogram (ECG) monitoring can also assist in the diagnosis of a PE. The ECG pattern that indicates a PE is an S1Q3T3 pattern with right ventricular strain. This pattern consists of an S wave in lead I, a Q wave in lead III, and an inverted T wave in lead III. Although this pattern is thought to be the most suggestive of PE for ECG findings, it is actually relatively rare and is only seen in less than 10% of EKGs in those with PE [8]. The most common ECG finding in PE diagnoses is actually sinus tachycardia.

Treatment for PE focuses on stabilizing the patient (e.g., fluids and oxygen), anticoagulation therapies, and adjunctive therapies to remove the blood clot if indicated. Initial anticoagulation should start immediately following diagnosis. In a hemodynamically stable patient, subcutaneous low-molecular-weight heparin, fondaparinux (Arixtra), or oral factor Xa inhibitors are the preferred anticoagulation agents [9]. In a hemodynamically unstable patient, unfractionated heparin is preferred due to its ability to be discontinued and reversed rather quickly [10]. Most patients with PE will need to have long-term anticoagulation therapy, usually the same medication as their initial therapy, to decrease the risk of another PE. This long-term maintenance therapy should be for at least three to six months following the initial PE but can last for up to 12 months [11]. The adjunctive therapies that are used to further dissolve and remove PEs include thrombolytic therapies (such as t-PA, a class of drugs that includes Activase), thrombectomies, and surgical options. A mechanical thrombectomy option involves the use of suction, allowing for the thrombus to be aspirated and removed via catheters. This option can reduce patients’ overall clot burden relatively quickly and significantly [12]. EkoSonic Endovascular System (EKOS) (Boston Scientific, Marlborough, MA, USA) is another type of adjunctive therapy that uses an ultrasound-guided catheter to deliver thrombolytic agents directly into the clot in small amounts over an extended time period [13]. This allows a smaller amount of thrombolytic medication to be given since it is placed directly in the clot.

## Case presentation

**Figure 1:**
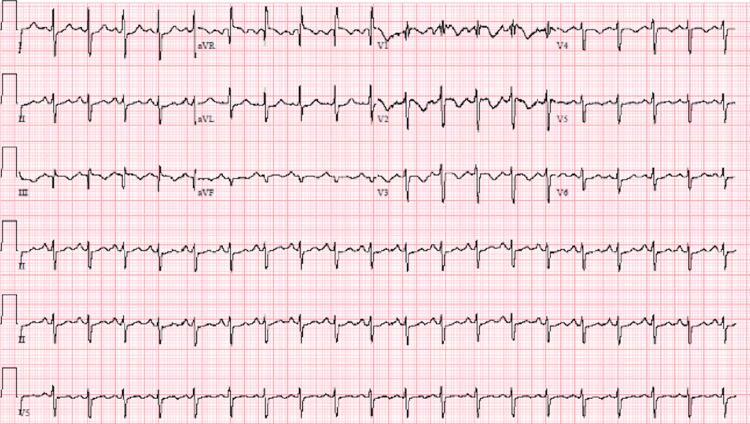
Electrocardiogram showing sinus tachycardia with ST segment and T wave abnormalities.

A 67-year-old male with no significant past medical history presented to the emergency department with a chief complaint of shortness of breath (SOB) beginning four days prior to arrival. The patient had received his second Pfizer COVID-19 vaccination two days prior to symptom onset. The SOB was first noted while he was completing yard work and did not resolve after 30 minutes of rest. His SOB continued with waxing and waning severity. On arrival to the ED, the patient was in minimal distress with tachypnea at a respiratory rate of 24 breaths/minute, and left lower extremity swelling was noted on physical examination. His physical examination was otherwise within normal limits. Creatinine was slightly elevated at 1.24 mg/dL, suggesting acute kidney injury. Additional abnormally elevated laboratory values included glucose of 291 mg/dL (for which a hemoglobin A1c was ordered), troponin I high sensitivity of 252.07 pg/mL, and brain natriuretic peptide (BNP) of 443.18 pg/mL. The remainder of the laboratory values were within normal limits. A 12-lead electrocardiogram (ECG) and a CT angiogram (CTA) of the chest with and without contrast were performed. The ECG showed normal sinus rhythm with 122 beats/minute, normal axis, normal intervals, and ST segment depression in lateral leads. T wave inversions were also noted over the anterior precordial leads. This impression is consistent with normal sinus tachycardia (Figure 1).

**Figure 2:**
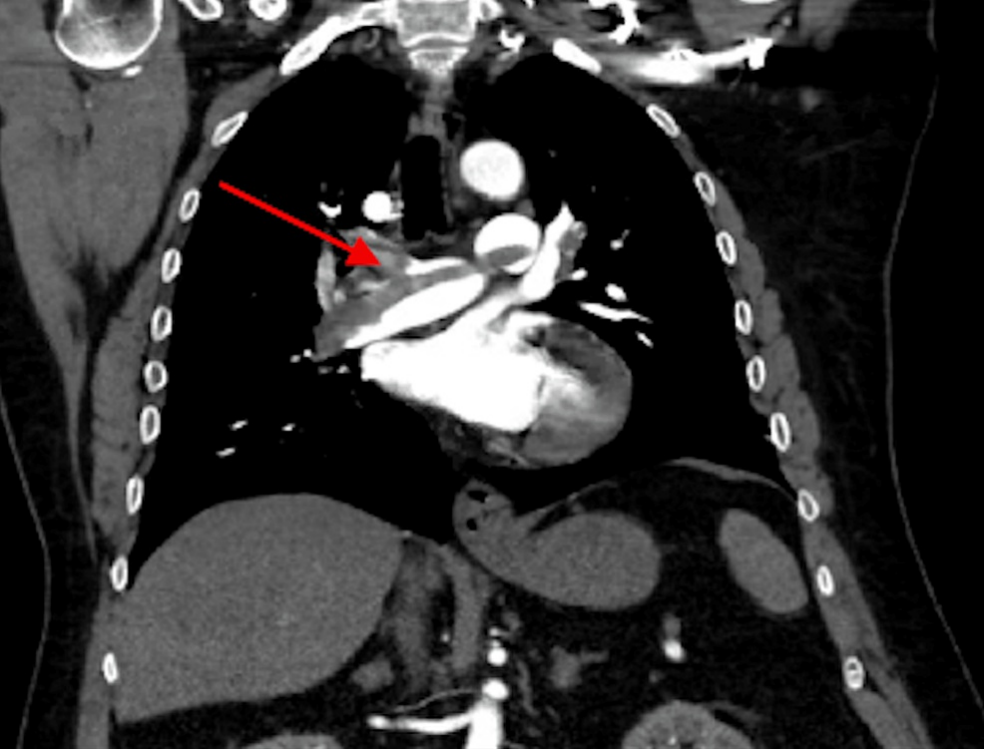
CT angiogram showing saddle pulmonary embolus (arrow).

The CTA showed a submassive saddle pulmonary embolism with right heart strain and a small hiatal hernia (Figure 2).

The patient was started on an IV heparin drip of 1,000 units per 500 mL of normal saline, admitted in critical condition, and immediately taken to the catheterization laboratory for either thrombectomy or EKOS. Catheter-assisted mechanical thrombectomy was successfully performed under local anesthesia. Normal systolic pulmonary artery pressure ranges from 18 to 22 mmHg, while diastolic pulmonary artery pressure normally ranges from 4 to 12 mmHg. Preoperative pulmonary artery pressure was 75/29 mmHg with a mixed venous saturation of 57%, while postoperative pulmonary artery pressure was 54/20 mmHg with a mixed venous saturation of 68%, demonstrating a significant improvement after thrombectomy.

**Figure 3:**
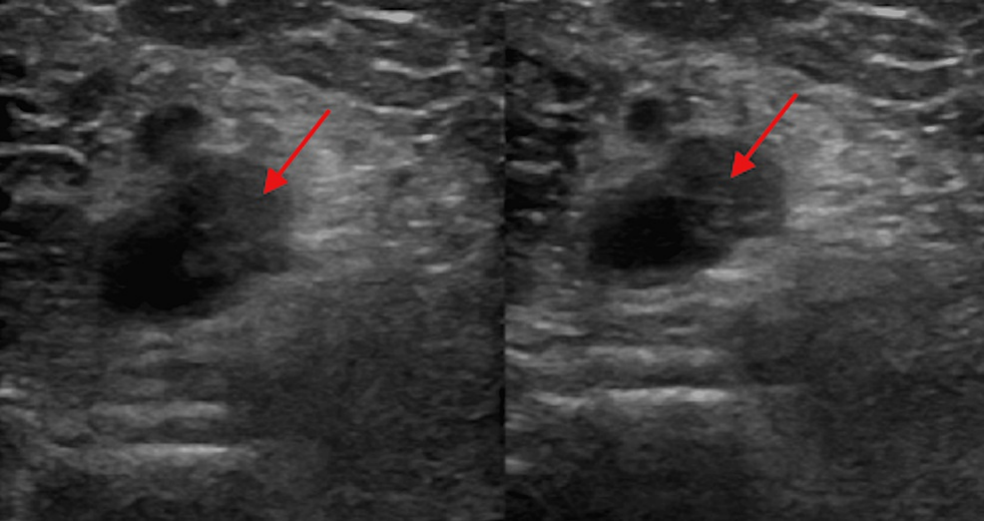
Doppler ultrasound showing intraluminal thrombus extending from the left distal popliteal vein to the left peroneal vein (arrows).

A cardiology consult was ordered by the ED team due to the CTA findings and the left lower extremity swelling. The cardiologist noted tachycardia, decreased breath sounds, SOB on minimal exertion, and a left lower extremity larger than the right lower extremity. Thus, an echocardiogram and a Doppler ultrasound of the left lower extremity were ordered and performed after admission to the hospital. The ultrasound showed a long intraluminal thrombus in the distal left popliteal vein extending into the calf and involving the left peroneal vein. This is referred to as deep vein thrombosis (DVT). This finding accounts for the difference in size between the lower extremities (Figure 3).

On postoperative day one (POD1), the patient reported notable improvement in his dyspnea following the thrombectomy, when evaluated by the hospitalist team. He still became short of breath when asked to sit up for pulmonary auscultation despite receiving oxygen via nasal cannula. The heparin drip was discontinued, and the patient was started on 10 mg of apixaban (Eliquis) twice a day for one week, followed by 5 mg twice a day. He was also given 40 mg atorvastatin (Lipitor) every night at bedtime along with continued intravenous hydration at 150 mL/hour. There was not a grossly visible discrepancy in size between the lower extremities, despite the confirmation of a left leg DVT.

On POD2, the patient was stable, reported no acute events overnight, and was weaned to room air. He was able to walk to the bathroom without experiencing any SOB or dizziness. His acute kidney injury improved to a creatinine value of 0.89 mg/dL. The hemoglobin A1c returned at 6.5%, suggesting that the patient was on the lower end of the range for the diagnosis of type 2 diabetes mellitus. He was started on 500 mg metformin (Glucophage) one tablet daily and counseled on glucose control with lifestyle management options to prevent further progression of his diabetes.

**Figure 4:**
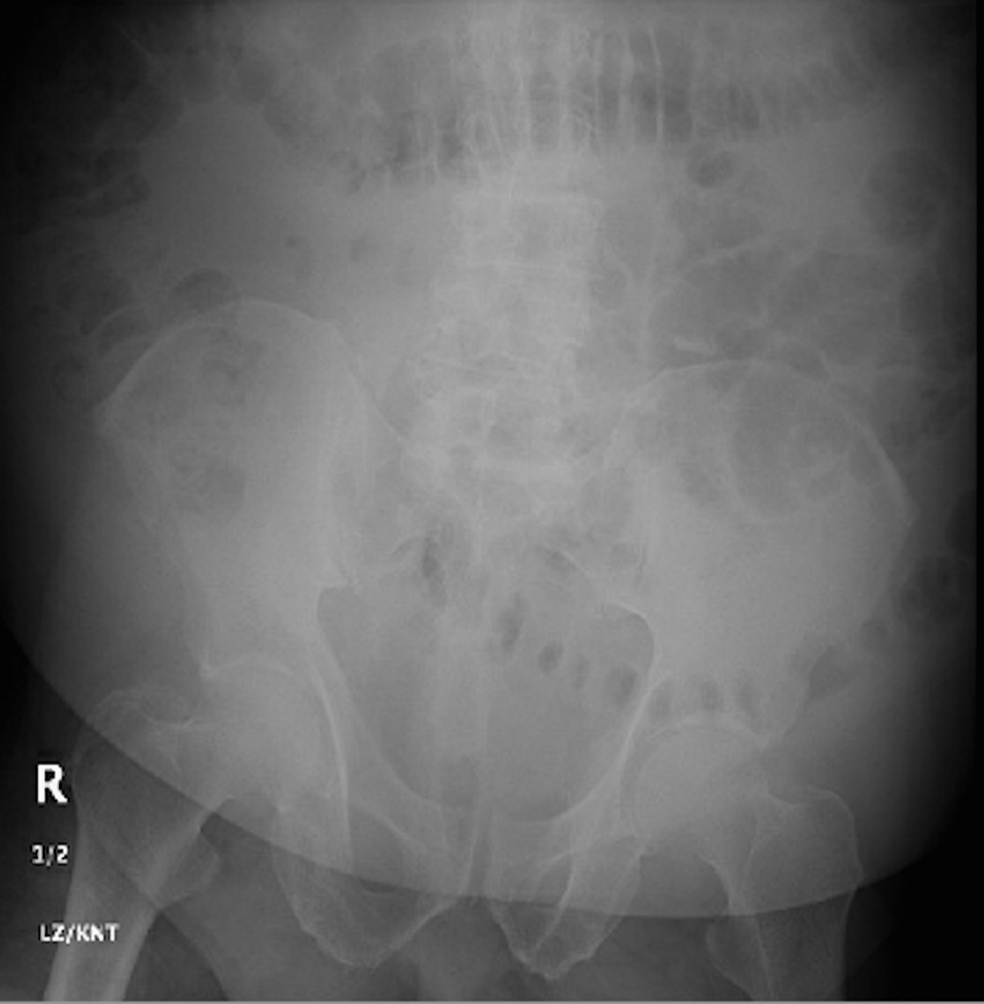
X-ray showing nonspecific bowel gas pattern with no evidence of obstruction and no evidence of any renal stones.

On POD3, the patient remained stable and continued to deny any dyspnea. He mentioned mild abdominal pain overnight that he described as similar to previous renal stones but stated it had since resolved. An X-ray of the abdomen showed no evidence of postoperative small bowel obstruction and did not identify any renal stones (Figure 4).

The patient was cleared for discharge at this point with instructions to continue the apixaban, atorvastatin, and metformin as previously ordered. He was also instructed to follow up with his primary care provider in three days and his cardiologist in one to two weeks.

## Discussion

Pulmonary emboli occur at a rate of 0.38/1,000 persons in the general population (0.038%) [14]. The severity and no known modifiable risk factors made this situation more unique than those seen with associated risk factors. This patient’s pulmonary embolism was classified as submassive (intermediate risk) because there was evidence of right ventricular strain on his CTA, but no hemodynamic instability. Right ventricular strain signifies an enlarged right ventricle with reduced systolic function secondary to increased pulmonary pressures from the blocked pulmonary vessels. The right ventricular strain was demonstrated via an enlarged right ventricle secondary to the right ventricular hypertension from the blocked pulmonary vessels. If he had not been treated as efficiently, he would have likely progressed to hemodynamic instability. At that point, his condition would have been classified as a massive pulmonary embolism (high risk).

Elevated BNP has limited diagnostic value in patients suspected of having PE. However, elevated BNP may be a valuable prognostic tool for risk stratification of patients diagnosed with acute PE [15]. In this patient, the BNP was elevated to more than four times the normal limit. Similarly, serum troponin I and T levels are useful prognostically, but not diagnostically. As markers of right ventricular dysfunction, troponin levels are elevated in 30%-50% of patients who have a moderate to large PE and are associated with clinical deterioration and death after PE [16]. This patient’s troponin I high sensitivity test being elevated at 252.07 pg/mL (normal: 45.2 pg/mL) provided further evidence of right ventricular strain and suggested a dismal outcome if urgent treatment had not been initiated.

The patient presented with the clinical symptoms of a DVT (unilateral leg swelling), imaging, a heart rate of >100 beats/minute (122 beats/minute), and laboratory values suggesting that other diagnoses were less likely than a pulmonary embolism. This presentation was awarded a 7.5 on the Wells criteria and allowed us to be confident of pulmonary embolism as the primary diagnosis prior to CTA. Once the diagnosis was confirmed via CTA, urgent treatment was indicated to prevent further decompensation. Due to the severity of this patient’s PE, thrombectomy was performed to provide rapid removal of the clot as systemic thrombolytic therapy was not indicated in this patient based on his presentation.

The patient did not possess any known risk factors prior to his symptomatic presentation. This classifies his PE as “unprovoked,” and although it was his first episode, it was recommended that he be placed on anticoagulant prophylaxis indefinitely rather than the usual three to six months of therapy to prevent recurrence. For patients with acute VTE, factor Xa inhibitors, such as apixaban, and direct thrombin inhibitors, such as dabigatran, are the usual first choice for oral anticoagulants for patients who need to be on long-term anticoagulation. This patient did not have any contraindications for these medications and was prescribed 10 mg apixaban twice daily for one week, followed by a change in dose to 5 mg twice a day afterward. The unprovoked character of this patient’s presentation also warranted follow-up ultrasonography of his left lower extremity two weeks after discharge to establish a new baseline and ideally confirm resolution of the DVT.

Vaccine-induced immune thrombotic thrombocytopenia (VITT) describes a prothrombotic syndrome some patients developed after receiving the COVID-19 vaccine [17]. Patients who developed VITT most notably had thrombosis of some type, elevated D-dimer, and occasionally thrombocytopenia [18]. These thromboses were most notably found within the deep veins of the leg, splanchnic vessels, and pulmonary arteries [19]. Thrombotic adverse events can be considered extremely rare after receiving the COVID-19 vaccine but have been reported for all three vaccine types [20]. An analysis of serious adverse events associated with COVID-19 vaccines was performed using data reports from Pfizer, Moderna, and Oxford-AstraZeneca. It found that there were 705 reports of pulmonary embolism as adverse events for all three vaccines combined, with 130 events for Moderna, 226 for Pfizer, and 349 for Oxford-AstraZeneca [20]. Overall, this analysis does support a relationship, although rare, between COVID-19 vaccines and thrombotic events.

## Conclusions

This case report highlights a case of an upper middle-aged male who was diagnosed with an unprovoked submassive saddle pulmonary embolism on CT angiogram and who was successfully treated with catheter-assisted mechanical thrombectomy and pharmacologic anticoagulation. Pulmonary emboli usually have common risk factors, such as obesity or recent immobilization, that physicians are trained to recognize to help aid in diagnosis. The possible relationship between COVID-19 vaccines and a prothrombotic state should be considered as a feasible new risk factor when patients present with possible pulmonary emboli but lack typical symptoms or risk factors.
